# Reduction of Orc6 Expression Sensitizes Human Colon Cancer Cells to 5-Fluorouracil and Cisplatin

**DOI:** 10.1371/journal.pone.0004054

**Published:** 2008-12-29

**Authors:** Elaine J. Gavin, Bo Song, Yuan Wang, Yaguang Xi, Jingfang Ju

**Affiliations:** 1 Cancer Genomics Laboratory, Mitchell Cancer Institute, University of South Alabama, Mobile, Alabama, United States of America; 2 Translational Research Laboratory, Department of Pathology, Stony Brook University, School of Medicine, Stony Brook, New York, United States of America; Ordway Research Institute, United States of America

## Abstract

Previous studies from our group have shown that the expression levels of Orc6 were highly elevated in colorectal cancer patient specimens and the induction of Orc6 was associated with 5-fluorouracil (5-FU) treatment. The goal of this study was to investigate the molecular and cellular impact of Orc6 in colon cancer. In this study, we use HCT116 (wt-p53) and HCT116 (null-p53) colon cancer cell lines as a model system to investigate the impact of Orc6 on cell proliferation, chemosensitivity and pathways involved with Orc6. We demonstrated that the down regulation of Orc6 sensitizes colon cancer cells to both 5-FU and cisplatin (cis-pt) treatment. Decreased Orc6 expression in HCT-116 (wt-p53) cells by RNA interference triggered cell cycle arrest at G1 phase. Prolonged inhibition of Orc6 expression resulted in multinucleated cells in HCT-116 (wt-p53) cell line. Western immunoblot analysis showed that down regulation of Orc6 induced p21 expression in HCT-116 (wt-p53) cells. The induction of p21 was mediated by increased level of phosphorylated p53 at ser-15. By contrast, there is no elevated expression of p21 in HCT-116 (null-p53) cells. Orc6 down regulation also increased the expression of DNA damaging repair protein GADD45β and reduced the expression level of JNK1. Orc6 may be a potential novel target for future anti cancer therapeutic development in colon cancer.

## Introduction

Orc6 is one of the six origin recognition complex protein in human cells. It functions as the initial assembly platform that is required for DNA replication. The roles of Orc6 in DNA replication have been investigated extensively in both yeast and Drosophila [1], [2], [3], [4], [5], [6]. But there are limited information on human cancer [7], [8]. It has been reported that in addition to its function as a DNA replication initiator protein, it also plays a key role in transcriptional gene silencing and heterochromatin formation [8]. However, it is not clear during the initiation complex assembly at which step that Orc6 participates [9]. It has been demonstrated elegantly by Prasanth *et al.* the dynamics of Orc6 localization during the entire cell cycle, including DNA replication, chromosome regregation, and cytokinesis. They also suggest Orc6 may function in signaling to cell cycle control [8]. Our interest in Orc6 stemmed from our previous studies with a comprehensive genomics analysis revealed that Orc6 is associated with 5-FU associated resistance in human colon cancer cell lines [10]. Our follow up study using colorectal patient samples further confirmed that the expression of Orc6 is highly elevated in human colorectal tumor tissues compared to paired normal specimens [11]. These results confirmed the clinical relevance of Orc6 leading to our hypothesis that Orc6 may be a key player that is involved in colorectal cancer. Its elevated level may also be a significant contributor to chemoresistance. p53 is one of the most frequently altered tumor suppressors in colorectal cancer and due to its critical function in cycle control [12], [13]. In this study, we use a pair of colon cancer cell lines with either wild type p53 or null p53 as our model system.

In this study, we provide experimental evidence that decreasing Orc6 expression by RNA interference can sensitize colon cancer cell lines to two of the major chemotherapeutic agents 5-FU and cisplatin treatment. Decreasing Orc6 expression by siRNA knock-down triggered cell cycle arrest and decreased cellular proliferation in HCT-116 (wt-p53) cells. By contrast, this effect was significantly impaired in HCT-116 (null-p53) cells. Alteration of cell cycle control by decreased Orc6 expression was due to the induction of p21, a major cell cycle control gene. The induction of p21 was due to the increased the level of phosphorylatd of p53 at ser-15 triggered by knock-down of Orc6 expression. Orc6 may be a potential novel target for new anti tumor therapeutic development.

## Results and Discussion

### Decreasing Orc6 expression in HCT-116 (wt-p53) cells inhibits cell proliferation

**Figure 1:**
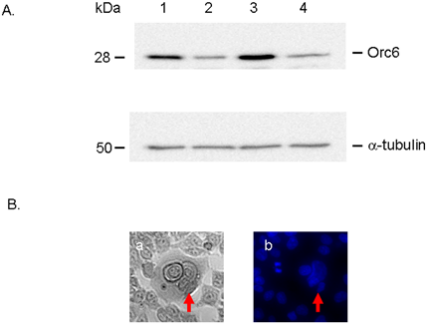
Decreased Orc6 expression by siRNA treatment using Western Immunoblot analysis in colon cancer cell line HCT116 (wt-p53) (lane 1, non-specific control; lane 2, specific siRNA for Orc6) and HCT116 (null-p53) cell line (lane 3 non-specific control; lane 4, specific siRNA for Orc6). α-tubulin was used as control (A). Increased multinucleation upon silencing of Orc6 in HCT-116 (wt-p53) cells by siRNA. Left panel, light image; Right panel, DAPI nuclear staining (B).

The increased levels of Orc6 in human colon cancer specimens led us to investigate the roles of Orc6 in cell cycle control and drug sensitivity. Using a siRNA knock-down approach, we first confirmed that the protein levels of Orc6 was decreased using Western immunoblot analysis in both HCT (wt-p53) cells (Figure 1A, lane 1, non-specific control siRNA; lane 2, siRNA against Orc6) and HCT-116 (null-p53) cells (Figure 1A, lane 3, non-specific control siRNA; lane 4, siRNA against Orc6). The expression of Orc6 protein was reduced by more than 5-fold in HCT (wt-p53) cells and HCT-116 (null-p53) cells.

**Figure 2:**
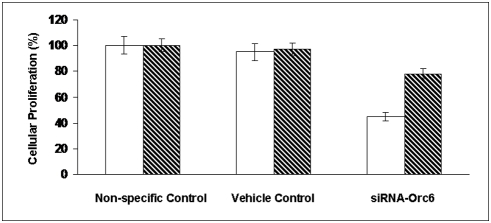
Effect of Orc6 on cell proliferation in human colon cancer HCT-116 (wt-p53) cells (open bar) and HCT116 (null-p53) cells (dashed bar).

**Figure 3:**
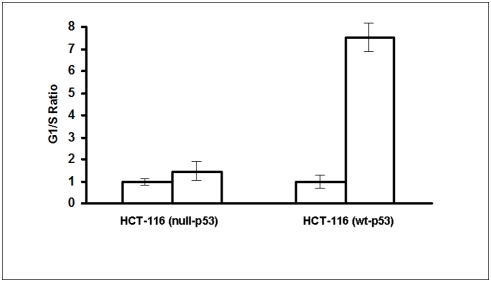
Effect of reduced Orc6 expression in G1/S cell cycle checkpoint control in both HCT-116 (wt-p53) and HCT (null-p53) cells by flow cytometry analysis.

It has been demonstrated previously that knock-down Orc6 in Hela cells induced polypoidy [8]. Our results confirmed this in colon cancer cells, after repeated transfection every 3 days, HCT-116 (wt-p53) cells with reduced Orc6 expression became multinucleated (Figure 1B). This population increased with time. Chromatin was stained with 4′,6′-diamidino-2-phenylindole (DAPI) to show multinucleated cells after sustained Orc6 knockdown by siRNA (left panel, light scatter cell image; right panel, DAPI nuclear staining image in Figure 1B). Cell proliferation rate was reduced by over 50% (open bar) with Orc6 knock-down (Figure 2). However, reduction of Orc6 expression in HCT-116 (null-p53) cells reduced in cell proliferation by only 23% (dashed bar). To investigate the impact of decreased Orc6 in cell cycle control, HCT-116 (wt-p53) and HCT-116 (null-p53) cells were first transfected with 100 nM Orc6 siRNA. Transfected cells were exposed to 10 µM 5-FU for 12 hrs. We observed that 12 hr later, both control HCT-116 (wt-p53) cells and cells with reduced Orc6 expressing cells were able to remain largely in the G1 phase without cell cycle re-entry (Figure 3A). By contrast, HCT-116 (null-p53) control cells and Orc6 reduced cells were re-entered into S phase of the cell cycle despite the DNA damage by 5-FU and Orc6 reduction (Figure 3B). These results suggest that the cell cycle control after DNA damage is depending on the presence of wild type p53.

### Effect of Orc6 on chemosensitivity to 5-FU and cis-pt

**Figure 4:**
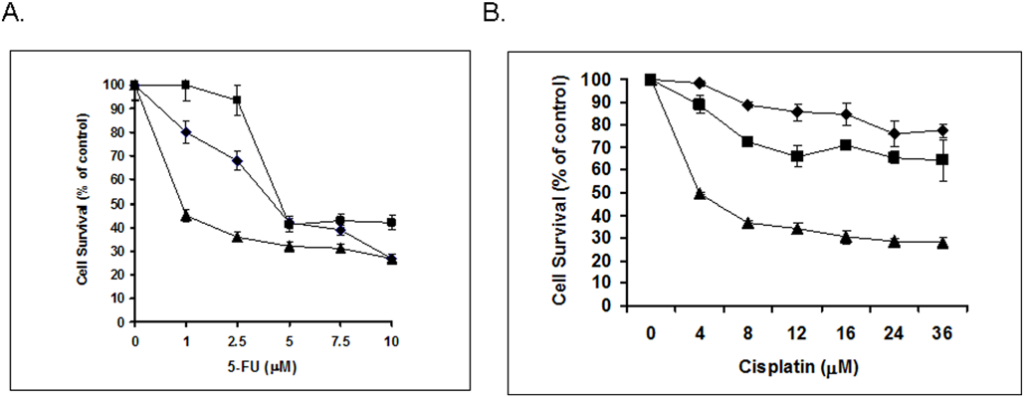
Effect of Orc6 expression on cytotoxicity of 5-FU and cis-pt in HCT-116 (wt-p53) cells.

To investigate the potential impact of Orc6 on chemosensitivity to some of the first line chemotherapeutic compounds such as 5-FU and cis-pt in colorectal cancer, HCT-116 (wt-p53) cells were used as our model system using cells transfected with oligofectamine alone, non-specific siRNA, and Orc6 specific siRNA. Cells then treated with 5-FU or cisplatin with serial dilution. After 72 hrs, cell proliferation was quantified by WST-1 assay. Figure 4A showed that the HCT-116 (wt-p53) cells with reduced Orc6 expression were 5-fold more sensitive to 5-FU treatment compared to control cells based on IC_50_ value. HCT-116 (wt-p53) cells with reduced Orc6 expression were also more sensitive to cisplatin treatment compared to control cells (Figure 4B). This effect was largely missing from HCT116 (null-p53) cells (data not show).

### Down stream signaling pathways effected by Orc6

**Figure 5:**
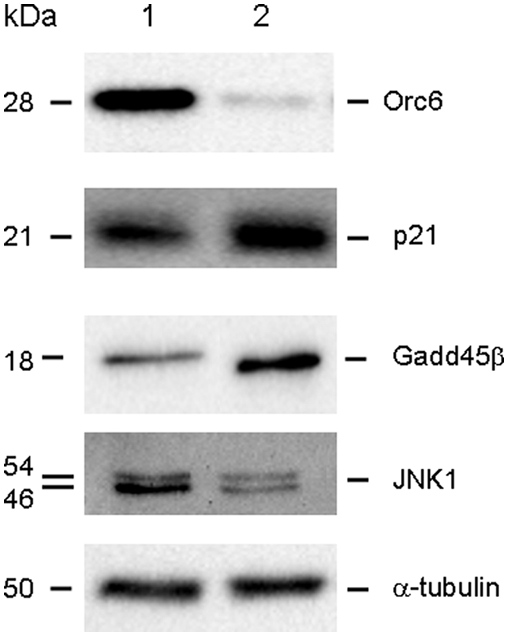
Western immunoblot analysis of cell cycle checkpoint control genes (p21, Gadd45β, and JNK1) affected by Orc6 knock-down in HCT-116 (wt-p53) cells.

**Figure 6:**
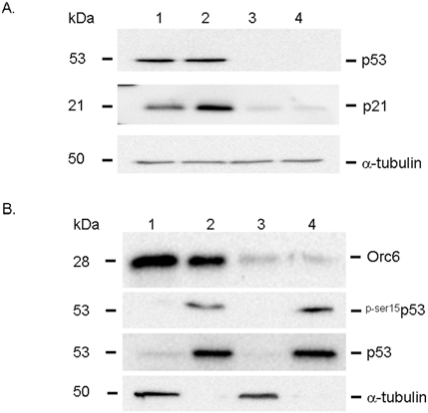
(A) Western immunoblot analysis of p53 and p21 expression after Orc6 knockdown by siRNA in both HCT-116 (wt-p53) cells (lane 1, non-specific control; lane 2, specific siRNA for Orc6) and HCT-116 (null-p53) cells (lane 3, non-specific control; lane 4, specific siRNA for Orc6). (B) Western immunoblot analysis of phosphorylated p53 expression at ser-15 in both cytoplasmic and nuclear fraction in control HCT-116 (wt-p53) cells (lane 1, cytoplasmic; lane 2, nuclear) and HCT-116 (wt-p53) cells treated with siRNA against Orc6 (lane 3, cytoplasmic; lane 4, nuclear). Total p53 protein expression levels were used as control protein in nuclear fractions and α-tubulin was used as control for cytoplasmic fractions.

To begin to understand the down stream signaling pathways potentially involved with Orc6, a high throughput gene expression analysis was used to quantify gene expression changes between HCT-116 (wt-p53) cells transfected with Orc6 specific siRNA and non-specific siRNA control. Expression and GeneOntology analysis showed that a number of genes were influenced by Orc6 inhibition (Data, S1). Based on our results, a number of well known cell cycle control genes were confirmed using Western immunoblot analysis. Our results showed that the expression of p21 was significantly induced in HCT-116 (wt-p53) cells after Orc6 knock-down (Figure 5, lane 1, non-specific oligo control; lane 2, siRNA of Orc6). We further confirmed that the expression of DNA damage repair protein Gadd45β was also induced by decreasing Orc6 expression in HCT-116 (wt-p53) cells (Figure 5, lane 1, non-specific siRNA control; lane 2, Orc6 specific siRNA). It is well documented that Gadd45β plays an important role in cell cycle arrest, DNA repair, innate immunity, maintenance of genome stability and apoptosis [7], [14], [15], [16]. The function of Gadd45β is mediated through interactions with other proteins such as cdc2 by disrupting the interaction between ccd2 and cyclin B1 in triggering G2/M cell cycle arrest under genotoxic stress [17], [18]. Our results showed that Gadd45β is at least, in part, responsible for the DNA replication defect triggered by reduced Orc6 expression in colon cancer cells. The expression of JNK1 level was decreased with reduced Orc6 expression (Figure 5). It has been shown that the roles of JNK1 were quite complex due to its due role in both apoptotic and survival signaling pathways [19], [20]. Fibroblasts with JNK knockouts are more sensitive to TNF-induced apoptosis [21]. Mouse embryo fibroblasts that lack MKK7 (upstream activator of JNK) undergo cellular senescence and G2/M growth arrest, further support our finding that reduced expression of JNK1 may be one of the contributing factors for G2/M arrest caused by knock-down of Orc6 expression [22]. These changes were largely absent from the HCT-116 (null-p53) cells (data not show). This is consistent with previous reported studies that p53 plays key role in cell cycle control in response to DNA damage. With the induction of p21 expression, we anticipate that the subsequent expression of p53 may be increased because p21 is a known cell cycle check point control gene mediated by p53. However, the total cellular level of p53 protein was not changed with the knock-down of p53 expression (Figure 6A, lane 1 and 2). The induction of p21 was absent in HCT-116 (null-p53) cells with Orc6 knock-down (Figure 6A, lane 3, control; lane 4, siRNA of Orc6). This indicates that the induction of p21 expression has to be p53 dependent despite the absence of in the p53 expression level. Although the total p53 level did not increase in HCT-116 (wt-p53) cells, we speculate that the level of p53 in the nuclear fraction may be increased due to known p53 translocation event. By separating nuclei and cytoplasm, we demonstrated that the level of total p53 protein in the nuclei was not elevated in HCT-116 (wt-p53) cells with reduced Orc6 expression treated by Orc6 specific siRNA (Figure 6A). Instead, the level of phosphorylated p53 at ser-15 was increased in the nuclear fraction of HCT-116 (wt-p53) cells with reduced Orc6 expression (Figure 6B). This is highly consistent with a well documented mechanism of p53 in response to DNA damage (14). Phosphorylated p53 at ser-15 residue can decrease its interaction with the negative regulator, the oncoprotein MDM2.

It seems that DNA damage response caused by a decreased Orc6 level is mediated through p53 dependent cell cycle control pathway. We speculate that high level of Orc6 in colorectal cancer may contribute to genome instability and perhaps by accumulated miss-firing of DNA replication with p53 deletions/mutations in over 50% of colorectal tumors. Loss of p53 in colorectal tumors further contributes to genome instability and accumulated mutations. Future studies will be needed to fully understand the molecular and cellular mechanism of Orc6 in colorectal cancer. Despite its essential role in the initial assembly platform required for DNA replication, Orc6 may have a potential as an novel target for anticancer therapeutic development. The situation maybe similar to 26S proteasome as a target for the antibody drug bortezomib. Initially it was thought that targeting 26S proteasome is not a good strategy due to its ubiquitous role in protein degradation [23]. Most recent report from Chen *et al.* suggests that Orc6 is dispensable in the DNA binding activity of Orcs in yeast. This further supports our notion that targeting Orc6 in colorectal cancer is a feasible strategy for future therapeutic development [24].

In conclusion, we demonstrated, in this study, that down regulation of Orc6 in colon cancer cells sensitize cells to 5-FU and cisplatin treatment. Together with our previous studies that Orc6 was highly overexpressed in colorectal cancer patients, we believe that Orc6 may have potential as a novel anticancer target for future anti-tumor therapeutic development.

## Materials and Methods

### Cell Culture

The human colon cancer cell lines, HCT-116 (wt-p53) and HCT-116 (null-p53) cells were a gift from Professor Bert Vogelstein (The Johns Hopkins University, Baltimore, MD), and were maintained in McCoy's 5A medium (Gibco Laboratories). 5-FU and cisplatin were purchased from Sigma.

### siRNA Transfection

HCT-116 (wt-p53) and HCT-116 (null-p53) were plated in 6-well trays at 1×10^5^cells/well and transfected with oligofectamine, non-specific control siRNA or siRNA against Orc6 at 100 nM concentration based on the manufacturer's protocol (Invitrogen Inc.)

### RNA Isolation

Total RNA was isolated from cell lines by using TRIzol reagent (Invitrogen, San Diego, CA) according to the manufacturer's instructions at 24 h after transfection with siRNA.

### Western immunoblot analysis

Cells were scraped and lysed in RIPA buffer (Sigma). Equal amounts of protein samples (50 µg) were resolved by SDS-PAGE on 12% gels by the method of Laemmli, and transferred to polyvinylidene fluoride membranes (PVDF) (BIO-RAD Laboratories). The membranes were then blocked by 5% nonfat milk in TBS-T (Tris-buffered saline and 0.5% Tween-20) at room temperature for 1 h. Proteins were probed with rat anti-orc6 monoclonal antibody (1∶1000 dilution, Upstate Technology), mouse anti-α-tubulin (1∶1000 dilution; Santa Cruz Biotechnology, Santa Cruz,Ca), mouse anti-p53 (1∶1000, Santa Cruz) followed by incubation with a horseradish peroxidase–conjugated secondary antibody (1∶1,000 dilution, Bio-Rad, Hercules, CA). Proteins were visualized with a chemiluminescence detection system using the Super Signal substrate (Pierce, Rockford, IL)

### Real time qRT-PCR analysis

cDNA synthesis was carried out with the High Capacity cDNA synthesis kit (Applied Biosystems) using 1 µg of total RNA as template. The PCR master mix containing TaqMan 2× Universal PCR Master Mix (No Amperase UNG), 10× TaqMan assay and RT products in 25 µl volume were processed as follows: 95°C for 3 min, followed by 40 cycles of 95°C for 15 sec and 60°C for 35 sec (n = 3). Signal was collected at the endpoint of every cycle. The gene expression 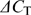 values of Orc6 from each sample were calculated by normalizing with internal control GAPDH and relative quantitation values were plotted.

### Cell cycle analysis by flow cytometry

HCT-116 (wt-p53) and HCT-116 (null-p53) were plated in 6-well trays at 1×10^5^cells /well and transfected with oligofectamine, non-specific control siRNA or Orc6 gene specific siRNA at 100 nM concentration. Cells were treated with 5-FU (10 µM) 12 hrs and cells were harvested by trypsin, washed and labeled with propidium iodide, washed and resuspended in Krisham's Modified buffer and filtered for flow cytometry analysis (BD FACS Caliber).

## Supporting Information

Data S1 GeneOntology. Gene Expression and GeneOntology analysis of differentially expressed genes in control and Orc6 knock-down HCT116 (wt-p53) cells.
(2.77 MB TIF)
