# Supplementary figures and images for: Reduction of Orc6 Expression Sensitizes Human Colon Cancer Cells to 5-Fluorouracil and Cisplatin
Source: PLoS One. 2008 Dec 29;3(12):e4054. doi: 10.1371/journal.pone.0004054 (PMC2603583; doi:10.1371/journal.pone.0004054)

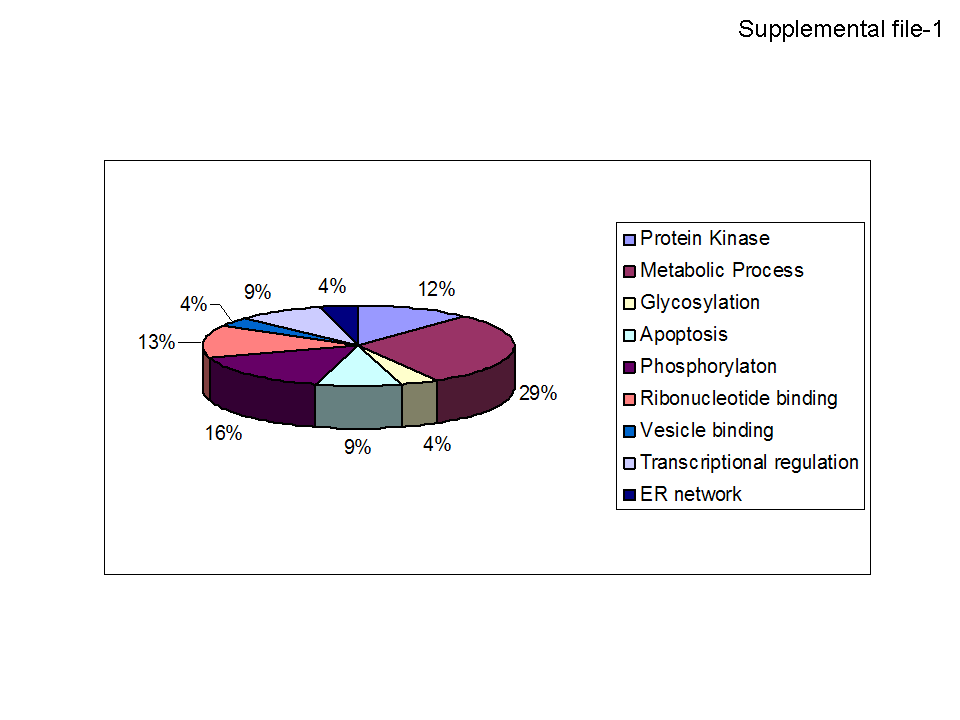

Supplement: Data S1 — GeneOntology. Gene Expression and GeneOntology analysis of differentially expressed genes in control and Orc6 knock-down HCT116 (wt-p53) cells. (2.77 MB TIF) [file pone.0004054.s001.tif]
